# A SINS/SRS/GNS Autonomous Integrated Navigation System Based on Spectral Redshift Velocity Measurements

**DOI:** 10.3390/s18041145

**Published:** 2018-04-09

**Authors:** Wenhui Wei, Zhaohui Gao, Shesheng Gao, Ke Jia

**Affiliations:** 1 School of Automatics, Northwestern Polytechnical University, 710072 Xi’an, China; alexandergao@mail.nwpu.edu.cn (Z.G.); gshshnpu@nwpu.edu.cn (S.G.); jk@nwpu.edu.cn (K.J.); 2 School of Geological Engineering and Surveying and Mapping, Chang’an University, 710064 Xi’an, China

**Keywords:** spectral redshift, autonomous navigation, integrated navigation, robust adaptive filtering, particle filtering

## Abstract

In order to meet the requirements of autonomy and reliability for the navigation system, combined with the method of measuring speed by using the spectral redshift information of the natural celestial bodies, a new scheme, consisting of Strapdown Inertial Navigation System (SINS)/Spectral Redshift (SRS)/Geomagnetic Navigation System (GNS), is designed for autonomous integrated navigation systems. The principle of this SINS/SRS/GNS autonomous integrated navigation system is explored, and the corresponding mathematical model is established. Furthermore, a robust adaptive central difference particle filtering algorithm is proposed for this autonomous integrated navigation system. The simulation experiments are conducted and the results show that the designed SINS/SRS/GNS autonomous integrated navigation system possesses good autonomy, strong robustness and high reliability, thus providing a new solution for autonomous navigation technology.

## 1. Introduction

At present, navigation methods mainly include inertial navigation, surface radio navigation, celestial navigation and satellite navigation. Each of these methods possess their own characteristics, scope of application and limitations, and cannot achieve completely autonomous or high precision autonomous navigation [1,2].

Strapdown inertial navigation system (SINS) has the advantages of simple structure and strong autonomy, providing continuous support of position, velocity and attitude information, under all-weather conditions. However, the navigation errors of SINS accumulate with time, thus the SINS cannot obtain high precision navigation information [3,4]. Radio navigation is limited by the coverage area of ground stations. The operation of this system is related to the radiowave propagation conditions, and vulnerable to the influence of artificial interference, which make radio navigation a non-autonomous navigation method [5,6,7]. Satellite navigation is the combination of celestial navigation and radio navigation, with the advantages of convenient application and high precision. However, because the satellite navigation depends on artificial beacons, it is also vulnerable to the influence of artificial interference and cannot achieve fully autonomy [8,9,10]. The advantages of celestial navigation are high accuracy of attitude measurement and strong ability to resist electromagnetic interference. Its disadvantages are the low rate of data updating, indirectly measured velocity and the limited navigation performance due to the number, distance and space environment of target celestial bodies [1,2,11,12].

The spectral redshift of natural light sources contains the velocity information of the celestial body relative to the moving object [13]. Based on this principle, spectral redshift navigation (SRS) becomes a forward-looking navigation method, with the advantages of simple principle, high navigation accuracy, strong autonomy and good real-time performance. The SRS can provide a new technological method to improve the autonomy of navigation systems. However, when the carrier is in the process of attitude maneuvering, the navigation accuracy will be worse and the navigation result may even be divergent due to the insufficient or interrupted observation information. Therefore, it is necessary to combine SRS with some other navigation method to constitute an integrated navigation system, thus compensating for the defects of SRS alone.

Nonlinear filtering algorithms are commonly used in autonomous navigation systems, however, these filtering algorithms have their own defects. For example, when the practical probability function has multiple peak values, the extended Kalman filtering (EKF) may be divergent because the nonlinear system equations arelinearized by the Taylor expansion and the linearized states are required to obey the Gaussian distribution [14,15,16,17]. The unscented Kalman filtering (UKF) method also demands the states obey the Gaussian distribution, which is not applicable for nonlinear systems with non-Gaussian distribution [18,19]. The particle filtering (PF) method is prone to particle degeneracy phenomena, and the accuracy depends heavily on the choice of importance sampling density and resampling scheme [20,21,22,23,24]. By robustly estimating the covariance matrix of observation noise and adaptively adjusting the covariance matrix of the state noise by augmenting the adaptive factor into the covariance matrix of the state prediction, the robust adaptive filtering can deal with observation and model noises to obtain reliable filtering results, especially in the presence of abnormal observations [25,26,27,28]. Therefore, combined with robust adaptive filtering and particle filtering, a new nonlinear filtering algorithm for autonomous navigation system is designed to improve the accuracy and reliability of the autonomous navigation system.

Based on the principle of velocity measurement by using spectral redshift of celestial bodies in space, and combined with the advantages of Geomagnetic Navigation System (GNS), this paper proposes a new SINS/SRS/GNS autonomous integrated navigation system. The principle, scheme and mathematical model of this autonomous integrated navigation system are established, and a high-precision nonlinear filtering algorithm for the autonomous navigation system is proposed. Subsequently, all of the models and algorithms are verified by experiments. 

## 2. The Principle of the Spectral Redshift Navigation

We assume the carrier can receive optical signals from celestial bodies during the space flight process. According to the Doppler effect principle, the spectral frequency of carrier received signals is not equal to the spectral frequency of the celestial bodies, and the variation of spectral frequency is related to the motion state of the carrier relative to the celestial bodies. Therefore, the relative velocity of the carrier can be obtained by measuring the redshift of the spectral frequency. According to the space vector relation, if the number of observed celestial bodies (which are non-collinear) is greater than three, the velocity vector of the carrier in inertial coordinate system can be determined by integrating the ephemeris of the celestial bodies and the inertial attitude information, and then the position vector can be obtained by integration.

The total redshift measured by spectral sensors is actually the sum of gravitational redshift, cosmological redshift and Doppler redshift. The gravitational redshift and cosmological redshift can be obtained by astronomical ephemeris, and both of them need to be removed. Only the Doppler redshift can be used to navigation calculation. We define the Doppler redshift as:

(1) $$z=\frac{\lambda -\lambda_{0}}{\lambda_{0}}=\frac{f_{0}-f}{f}$$

where z represents Doppler redshift, $\lambda_{0}$ is the original wavelength of the spectral line, λ is the observed wavelength, $f_{0}$ is the original frequency of the spectral line, *f* is the observed frequency.

Accordingly, the redshift equation is:

(2) 1+z=f0f=1+vcosθ/c1−v2c2

where v is the velocity of the carrier relative to the light source, θ is the angle between the wave vector of the light source pointing to the carrier and the velocity v in the inertial coordinate system, vcosθ represents radial velocity, c is the light speed in vacuum.

In three-dimensional celestial navigation, Equation (2) is transformed and applied to the first reference celestial body, and we can obtain:

(3) $$v_{r1}=\left(1+z_{1}\right)\sqrt{c^{2}-{\left|v_{p}-v_{1}\right|}^{2}}-c$$

where $v_{r1}$ is the radial velocity of the carrier relative to the first reference celestial body (the light source), $z_{1}$ is the redshift value of the first reference celestial body, $v_{p}$ is the velocity vector of the carrier in the inertial coordinate system, $v_{1}$ is the velocity vector of the first reference celestial body in the inertial coordinate system. The redshift value can be obtained by spectral preprocessing, spectral line feature extraction and spectral line matching.

Select three reference celestial bodies and the equations are listed as follows:

(4) $$\left\{ \begin{array}{l} v_{r1}=\left(1+z_{1}\right)\sqrt{c^{2}-{\left|v_{p}-v_{1}\right|}^{2}}-c\\ v_{r2}=\left(1+z_{2}\right)\sqrt{c^{2}-{\left|v_{p}-v_{2}\right|}^{2}}-c\\ v_{r3}=\left(1+z_{3}\right)\sqrt{c^{2}-{\left|v_{p}-v_{3}\right|}^{2}}-c \end{array}\right.$$

From the geometric relationship of the celestial bodies, the relationship between $v_{P}$ and $v_{r1}$, $v_{r2}$, $v_{r3}$ satisfies the following relationship:

(5) $$\left\{ \begin{array}{l} v_{r1}=\left(v_{P}-v_{1}\right)\cdot \;u_{1}\\ v_{r2}=\left(v_{P}-v_{2}\right)\cdot \;u_{2}\\ v_{r3}=\left(v_{P}-v_{3}\right)\cdot \;u_{3} \end{array}\right.$$

where $v_{1}$, $v_{2}$ and $v_{3}$ are the velocity vectors of the celestial bodies in the inertial system determined by the ephemeris, respectively, $u_{1}$, $u_{2}$ and $u_{3}$ are the unit vectors of the position vectors of the celestial bodies pointing to the carrier in the inertial coordinate system, which can be measured by the sun sensor or the star sensor.

The state estimation equations for the velocity vector and the position vector are established as:

(6) $$\left\{ \begin{array}{l} v_{P}=f\left(v_{1},\;v_{2},\;v_{3},\;u_{1},\;u_{2},\;u_{3},v_{r1},\;v_{r2},\;v_{r3}\;\right)\\ r_{P}=\int v_{P}dt \end{array}\right.$$

After the initial value is given, the velocity vector $v_{P}$ of the carrier in the inertial coordinate system can be obtained by solving (6), and then the position vector $r_{P}$ can be obtained by integration.

## 3. Design of the SINS/SRS/GNS Autonomous Integrated Navigation System

**Figure 1:**
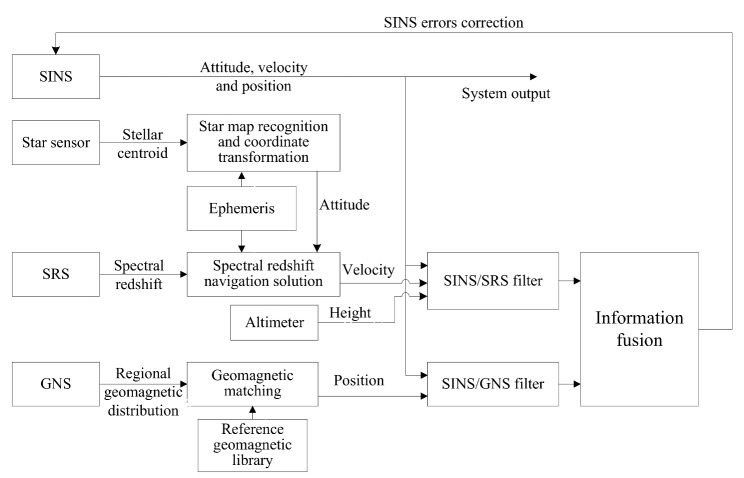
The principle of the SINS/SRS/GNS autonomous integrated navigation system.

Since the SINS can provide three-dimensional attitude, speed and position in all weather and all day conditions, and possesses the ability of good concealment and strong anti-interference. it is usually used as the main navigation system. SRS and GNS are used as auxiliary navigation systems to form the SINS/SRS/GNS autonomous integrated navigation system, as shown in Figure 1.

The estimation of spectral redshift can be calculated by de-noising and spectral lines separation of spectral signals that obtained by a spectrometer. According to the principle of spectral redshift navigation, the accurate velocity information of the carrier can be obtained by combining the celestial ephemeris and the attitude information measured by the star sensor. The difference between this velocity and the velocity obtained by SINS is made as observation vector and sent to the SINS/SRS subfilter for filtering calculation. GNS can get regional geomagnetic distribution by magnetic sensor, and the position information of the carrier can be obtained by matching the regional geomagnetic distribution and the reference geomagnetic library. The difference between this position and the position from SINS is treated as observation vector, which is sent to SINS/GNS subfilter for filtering calculation. The attitude errors, position errors and velocity errors calculated by the SINS/SRS subfilter and the SINS/GNS subfilter are both sent into the global filter for multi-sensor information fusion. Then the global optimal estimation of navigation errors is calculated to correct the SINS, for obtaining the accurate output of SINS/SRS/GNS autonomous integrated navigation system.

### 3.1. State Equation of the Autonomous Integrated Navigation System

Using the E-N-U (East-North-Up) geographic coordinate system as the base coordinate system for navigation. The system state vector x(t) of SINS/SRS/GNS autonomous integrated navigation system can be defined as:

(7) $$x(t)={\left[{\left(\delta q\right)}^{T},{\left(\delta V\right)}^{T},{\left(\delta P\right)}^{T},\varepsilon^{T},\nabla^{T}\right]}^{T}$$

where $\delta q={\left[\delta q_{0},\delta q_{1},\delta q_{2},\delta q_{3}\right]}^{T}$ is the attitude error quaternion of SINS, $\delta V={\left[\delta V_{E},\delta V_{N},\delta V_{U}\right]}^{T}$ is the velocity error of SINS, $\delta P={\left[\delta L,\delta \lambda ,\delta h\right]}^{T}$ is the latitude error, longitude error and height error of SINS, $\varepsilon ={\left[\varepsilon_{x},\varepsilon_{y},\varepsilon_{x}\right]}^{T}$ represents the random drift of the gyroscope, $\nabla ={\left[\nabla_{x},\nabla_{y},\nabla_{z}\right]}^{T}$ is the constant bias of the accelerometer.

According to (7) and the error model of SINS [10], the kinematic model of the SINS/SRS/GNS autonomous integrated navigation system is described as:

(8) $$\dot{x}=f(x,t)+G(t)w(t)$$

where f(x,t) is nonline state function of the system, G(t) is the coefficient matrix of system noise. $w(t)={\left[w_{gx},w_{gy},w_{gz},w_{ax},w_{ay},w_{az}\right]}^{T}$ represents system noise, $\left[w_{gx},w_{gy},w_{gz}\right]$ is the white Gaussian noise of the gyro measurement, $\left[w_{ax},w_{ay},w_{az}\right]$ is the white Gaussian noise of the accelerometer measurement:

(9) $$f(x,t)=\left[\begin{array}{l} B(I-C_{n}^{c})\omega_{in}^{n}-BC_{b}^{c}\varepsilon^{b}\\ (I-C_{c}^{n})C_{b}^{c}f^{b}-(2\omega_{ie}^{n}+\omega_{en}^{n})\times \delta V^{n}-(2\delta \omega_{ie}^{n}+\delta \omega_{en}^{n})\times V^{n}+C_{b}^{c}\nabla^{b}\\ M\delta V+N\delta L\\ 0_{3\times 1}\\ 0_{3\times 1} \end{array}\right]$$

$$G(t)=\left[\begin{array}{cc} C_{b}^{c} & 0_{3\times 3}\\ 0_{3\times 3} & C_{b}^{c}\\ 0_{3\times 3} & 0_{3\times 3}\\ 0_{3\times 3} & 0_{3\times 3}\\ 0_{3\times 3} & 0_{3\times 3} \end{array}\right]$$

where $C_{n}^{c}$ and $C_{b}^{c}$ are the attitude transition matrixes of the navigation coordinate system n and the carrier coordinate system b to the computing coordinate system c, respectively. $\omega_{in}^{n}$ represents the projection of the angular velocity of the carrier in the navigation coordinate system, $\omega_{ie}^{n}$ and $\omega_{en}^{n}$ are the rotation angle velocity and the position angular velocity of the earth, respectively. $V^{n}$ is the velocity of the carrier, $\nabla^{b}$ is the error of the accelerometer, $f^{b}$ is the accelerometer’s specific force. M and N are the coefficient matrixes of the velocity error and position error respectively, δ⋅represents the corresponding error of the parameter. $B=\frac{1}{2}M(Q_{p}^{n})$, $M(Q_{p}^{n})$ is the matrix representation of quaternion multiplication, and:

(10) $$M(Q_{p}^{n})=\left[\begin{array}{cccc} q_{0} & -q_{1} & -q_{2} & -q_{3}\\ q_{1} & q_{0} & -q_{3} & q_{2}\\ q_{2} & q_{3} & q_{0} & -q_{1}\\ q_{3} & -q_{2} & q_{1} & q_{0} \end{array}\right]$$

### 3.2. Observation Equation of the Autonomous Integrated Navigation System

(1) Observation equation of SINS/SRS subsystem

Let $v_{X}$, $v_{Y}$ and $v_{Z}$ represent the E-velocity, N-velocity and U-velocity of the carrier in the inertial system, respectively, which are obtained by the spectral redshift navigation, then:

(11) $$V_{n}=C_{b}^{n}V_{b}$$

(12) $$V_{n}=\left[\begin{array}{c} v_{SE}\\ v_{SE}\\ v_{SE} \end{array}\right],V_{b}=\left[\begin{array}{c} v_{X}\\ v_{Y}\\ v_{Z} \end{array}\right]$$

where $C_{b}^{n}$ is the attitude transition matrixes of the body coordinate system to the navigation coordinate system, $V_{n}$ is velocity vector of the carrier in navigation coordinate system, $V_{b}$ is velocity vector of the carrier in body coordinate system. $v_{SE}$, $v_{SN}$ and $v_{SU}$ are E-velocity, N-velocity and U-velocity of the carrier in navigation coordinate system, respectively, which are obtained by the spectral redshift navigation.

Take the difference of the velocity between SRS and SINS output as the observation, and the velocity observation equation is obtained as:

(13) $$Z_{v}=\left[\begin{array}{c} Z_{S1}\\ Z_{S2}\\ Z_{S3} \end{array}\right]=\left[\begin{array}{c} v_{E}-v_{SE}\\ v_{N}-v_{SN}\\ v_{U}-v_{SU} \end{array}\right]=H_{v}X\left(t\right)+V_{v}\left(t\right)$$

where $H_{v}$ is the velocity observation matrix, $v_{E}$, $v_{N}$ and $v_{U}$ are the E-velocity, N-velocity and U-velocity of the carrier, respectively, which are obtained by the SINS, $V_{v}$ is velocity observation noise. $H_{v}$ can be expressed as:

(14) $$H_{v}={\left[\begin{array}{ccc} 0_{3\times 3} & diag\left(\begin{array}{ccc} 1 & 1 & 1 \end{array}\right) & 0_{3\times 9} \end{array}\right]}_{3\times 15}$$

Accordingly, the observation equation of SINS/SRS subsystem can be expressed as:

(15) $$\begin{array}{ll} Z_{1}\left(t\right) & =\left[Z_{v}\left(t\right)\right]=\left[H_{v}\right]X\left(t\right)+\left[V_{v}\left(t\right)\right]\\ & =H_{1}\left(t\right)X\left(t\right)+v_{1}\left(t\right) \end{array}$$

where $H_{1}\left(t\right)$ is observation matrix, $V_{1}\left(t\right)$ is white noise vector.

(2) Observation equation of SINS/GNS subsystem

The difference between the positions of SINS and GNS is selected as the observation, and the observation vector can be described as:

(16) $$Z_{2}(t)=\left[\begin{array}{cc} \begin{array}{c} L_{I}\\ \lambda_{I}\\ h_{I} \end{array} & \begin{array}{c} L_{G}\\ \lambda_{G}\\ h_{G} \end{array} \end{array}\right]=\left[\begin{array}{c} \left(L+\delta L_{I})-(L+\delta L_{G}\right)\\ \left(\lambda +\delta \lambda_{I})-(\lambda +\delta \lambda_{G}\right)\\ \left(h+\delta h_{I})-(h+\delta h_{G}\right) \end{array}\right]=\left[\begin{array}{c} \delta L_{I}\\ \delta \lambda_{I}\\ \delta h_{I} \end{array}\right]-\left[\begin{array}{c} \delta L_{G}\\ \delta \lambda_{G}\\ \delta h_{G} \end{array}\right]$$

where $L_{I}$, $\lambda_{I}$ and $h_{I}$ are latitude, longitude, and height of the SINS output, respectively. *L_G_*, $\lambda_{G}$ and $h_{G}$ are the latitude, longitude, and height of the GNS, respectively. δ⋅represents the corresponding parameter error.

According to (18), the observation equation of the SINS/GNS subsystem can be obtained as:

(17) $$Z_{2}(t)=H_{2}(t)X(t)+V_{2}(t)$$

where $H_{2}(t)$ is the observation matrix, $V_{2}(t)={\left[\delta L_{G},\delta \lambda_{G},\delta h_{G}\right]}^{T}$ is a white noise vector.

### 3.3. Information Fusion Algorithm of the Autonomous Integrated Navigation

**Figure 2:**
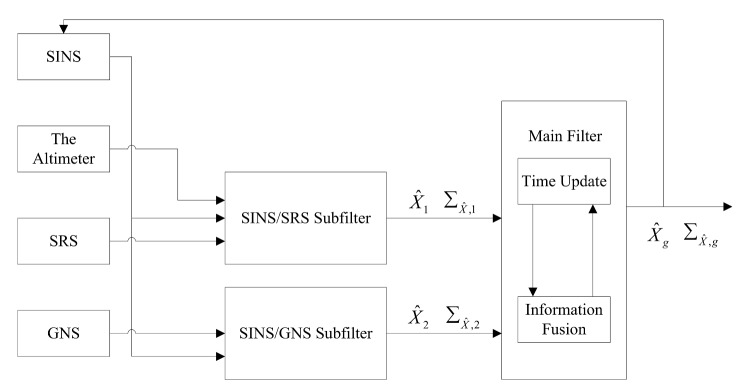
The information fusion principle of SINS/SRS/GNS autonomous integrated navigation system.

According to federal filtering technology [29,30], the information fusion principle of SINS/SRS/GNS autonomous integrated navigation system is shown in Figure 2. Firstly, SINS/SRS and SINS/GNS navigation subfilters are designed, and two sets of local optimal estimation ${\hat{X}}_{i}$ and local optimal error covariance matrix $\Sigma_{\hat{X},i}(i=1,2)$ are obtained. Secondly, the federated filtering technology is applied to send two sets of local optimal estimation into the main filter for information fusion, and the global optimal estimation ${\hat{X}}_{g}$ and the global optimal error covariance matrix $\Sigma_{\hat{X},g}$ of the system state vector are obtained. Finally, the SINS error is corrected in real time by using ${\hat{X}}_{g}$. Due to the divergence of the SINS height channel, the height of the altimeter output is used to correct the SINS. The optimal fusion algorithm for SINS/SRS/GNS autonomous integrated navigation system is expressed as:

(18) $$\{\begin{array}{c} \Sigma_{\hat{X},g}={\left(\Sigma_{\hat{X},1}^{-1}+\Sigma_{\hat{X},2}^{-1}\right)}^{-1}\\ {\hat{X}}_{g}=\Sigma_{\hat{X},g}(\Sigma_{\hat{X},1}^{-1}{\hat{X}}_{1}+\Sigma_{\hat{X},2}^{-1}{\hat{X}}_{2}) \end{array}$$

## 4. Robust Adaptive Central Difference Particle Filtering Algorithm

Combined with the advantages of robust adaptive filtering (RAF), central difference Kalman filtering (CDKF) and particle filtering (PF), a robust adaptive central difference particle filtering algorithm (RACDPF) is proposed. By choosing appropriate equivalent weight function and adaptive factor, the algorithm controls the information of the state model and observation model to suppresse the influence of abnormal interference on the system state estimation, and updates the a priori information, adjusts the filtering gain, for improving the filtering accuracy.

### 4.1. Algorithm Steps

Consider the nonlinear system model:

(19) $$\begin{array}{l} x_{k}=f(x_{k-1},v_{k-1})\\ y_{k}=h(x_{k},n_{k}) \end{array}$$

The main steps of the robust adaptive central difference particle filtering algorithm are as follows.

(1) Initialization. At the time k=0:
  (20) $$\begin{array}{l} {\hat{x}}_{0}=E[x_{0}]\\ P_{x_{0}}=E{\left[(x_{0}-{\hat{x}}_{0})(x_{0}-{\hat{x}}_{0})\right]}^{T} \end{array}$$
(2) When k=1,⋯,∞, and j=0,⋯,2L:
  (21) $$\begin{array}{l} W_{0}^{m}=\frac{h^{2}-L}{h^{2}},W_{j}^{m}=\frac{1}{2h^{2}}\\ W_{j}^{c1}=\frac{1}{4h^{2}},W_{j}^{c2}=\frac{h^{2}-1}{4h^{4}} \end{array}$$
  where L is state dimension, h represents the central differential interval of the scalar, for Gauss prior random variables, the optimal value is $h=\sqrt{3}$.
  Time update:
  (22) $$\chi_{k-1}^{i}=[{\hat{x}}_{k-1}^{i},{\hat{x}}_{k-1}^{i}+h\sqrt{P_{k-1}^{i}},{\hat{x}}_{k-1}^{i}-h\sqrt{P_{k-1}^{i}}]$$
  (23) $$\chi_{k|k-1}^{i}=f(\chi_{k-1}^{i},u_{k-1}^{i})$$
  (24) $${\hat{x}}_{k}^{i-}=\sum_{j=0}^{2L}W_{j}^{m}\chi_{j,k|k-1}^{i}$$
  (25) $$P_{x_{k}}^{i-}=\sum_{j=0}^{L}[W_{j}^{c_{1}}{\left(\chi_{j,k|k-1}^{i}-\chi_{L+j,k|k-1}^{i}\right)}^{2}+W_{j}^{c_{2}}{\left(\chi_{j,k|k-1}^{i}+\chi_{L+j,k|k-1}^{i}-2\chi_{0,k|k-1}^{i}\right)}^{2}]$$
  (26) $$\chi_{k|k-1}^{i*}=[{\hat{x}}_{k|k-1}^{i},{\hat{x}}_{k|k-1}^{i}+h\sqrt{P_{x_{k|k-1}}^{i-}},{\hat{x}}_{k|k-1}^{i}-h\sqrt{P_{x_{k|k-1}}^{i-}}]$$
  Observation update:
  (27) $$Y_{k|k-1}^{i}=h(\chi_{k|k-1}^{i*})$$
  (28) $${\hat{y}}_{k}^{i-}=\sum_{j=0}^{2L}W_{j}^{m}Y_{j,k|k-1}^{i}$$
  (29) $$P_{{\bar{y}}_{k}}^{i}=\sum_{j=0}^{L}[W_{j}^{c_{1}}{\left(Y_{j,k|k-1}^{i}-Y_{L+j,k|k-1}^{i}\right)}^{2}+W_{j}^{c_{2}}{\left(Y_{j,k|k-1}^{i}+Y_{L+j,k|k-1}^{i}-2Y_{0,k|k-1}^{i}\right)}^{2}]$$
  (30) $$P_{x_{k}y_{k}}^{i}=\sqrt{W_{1}^{{}_{c_{1}}}P_{x_{k}}^{i-}}{\left[Y_{1:L,k|k-1}^{i}-Y_{L+1:2L,k|k-1}^{i}\right]}^{T}$$
  The predicted residual vector contains the state information that is not corrected by observation information, and can reflect the disturbance of the dynamic system. Therefore, we can use the predicted residual vector as the variable to construct the error discriminant statistic and the adaptive factor of the kinematic model. The ith predicted residual vector ${\bar{V}}_{k}^{i}$ at time *k* can be expressed as:
  (31) $${\bar{V}}_{k}^{i}=Y_{k}^{i}-{\hat{y}}_{k}^{i}$$
  Correspondingly, the error discriminant statistic of the kinematic model is written as:
  (32) $$\Delta {\bar{V}}_{k}^{i}={\left(\frac{{\left({\bar{V}}_{k}^{i}\right)}^{T}{\bar{V}}_{k}^{i}}{tr(P_{y_{k}y_{k}}^{i})}\right)}^{\frac{1}{2}}$$
  Then, the adaptive factor that is based on the error discriminant statistic can be obtained as [25]:
  (33) $$\alpha_{k}^{i}=\left\{ \begin{array}{l} 1\;\left|\Delta {\bar{V}}_{k}^{i}\right|\leq c\;\\ \frac{c}{\left|\Delta {\bar{V}}_{k}^{i}\right|}\;\left|\Delta {\bar{V}}_{k}^{i}\right|>c \end{array}\right.$$
  where $\alpha_{k}^{i}$ is the ith adaptive factor at time k, c is an empirical constant, and generally 1.0<c<2.5.
  According to the Kalman filtering framework, we can obtain:
  (34) $$K_{k}^{i}=P_{x_{k}y_{k}}^{i}{\left(P_{{\bar{y}}_{k}}^{i}\right)}^{-1}$$
  (35) $${\hat{x}}_{k}^{i}={\hat{x}}_{k}^{i-}+K_{k}^{i}(y_{k}^{i}-{\hat{y}}_{k}^{i-})$$
  (36) $$P_{x_{k}}^{i}=P_{x_{k}}^{i-}-\alpha_{k}^{i}K_{k}^{i}P_{{\bar{y}}_{k}}^{i}K_{k}^{iT}$$
  It can be seen from (38) that the adaptive factor $\alpha_{k}$ can influence and adjust $P_{x_{k}}$ to make the importance density function closer to the actual distribution. When anomalies exist in the observation model, the adaptive factor $\alpha_{k}$ decreases, the use of observation information is reduced in the process of the state estimation, thus weakening the abnormal interference of the observation model, or vice versa.
  Let $N({\hat{x}}_{k}^{i},p_{x_{k}}^{i})$ be the importance density function of the particle sampling, the new sample $x_{k}^{i}∼N({\hat{x}}_{k}^{i},P_{x_{k}}^{i})$ can be obtained by importance sampling. In addition, compared with the UKF algorithm, the CDKF algorithm only needs to calculate the parameter h, therefore the computational complexity is reduced.
(3) Calculate the weight:
  (37) $$w_{k}^{i}=w_{k-1}^{i}\frac{p(y_{k}|x_{k}^{i})p(x_{k}^{i}|x_{k-1}^{i})}{q(x_{k}^{i}|x_{k-1}^{i},y_{k})}$$
  and normalize the weight to ${\tilde{w}}_{k}^{i}=w_{k}^{i}/\sum_{i=1}^{n}w_{k}^{i}$.
(4) Calculate the threshold estimate:
  (38) $${\hat{N}}_{eff}=1/\sum_{i=1}^{N}{\left({\tilde{w}}_{{}_{k}}^{i}\right)}^{2}$$
  Compare the result with the established threshold to determine the degree of the particle degradation. The smaller ${\hat{N}}_{eff}$ is, the worse the particle degeneracy is. In order to inhibit particle degeneracy, *M* new particles can be obtained by resampling the posterior density function, and given the same weight 1/*M*.
(5) Markov Chain Monte Carlo (MCMC) move. This step can be selected according to the needs.
(6) Calculate the estimate of the nonlinear state vector and its covariance matrix:
  (39) $${\hat{x}}_{k}^{}=\sum_{i=1}^{N}{\tilde{w}}_{k}^{i}x^{i}{}_{k}$$
  (40) $${\hat{P}}_{k}^{}=\sum_{i=1}^{N}{\tilde{w}}_{k}^{i}(x_{k}^{i}-{\hat{x}}_{k}^{}){\left(x_{k}^{i}-{\hat{x}}_{k}^{}\right)}^{T}$$
  return to Step (2).

In the above steps, the Expectation Maximization (EM) [23] method can be used to replace the resampling, so that the state estimation is converged to the optimal value. In addition, when selecting the importance density function, the proposed RACDPF takes advantage of the important adjustment factor, namely the robust adaptive factor, which controls the contribution of the observed information in the state estimation, and provides better sampling function for the importance sampling process.

### 4.2. Adaptive Adjustment of the Weight

In the RACDPF algorithm, the adaptive adjustment method of the weight can be used to calculate the weight value, for reducing the amount of calculation while improving the accuracy. The principle of the method is that after the steps of time update and observation update, utilize observation information, Euclidean distance and precision dilution, which reflects statistical characteristics of observation noise to Euclidean distance, to adaptively adjust the weight, thus move the sampled particles from the high priori density region to the high likelihood density region, to obtain a posteriori density function closer to the true distribution.

After sampling the importance density function $N({\bar{x}}_{k}^{i},P_{k}^{i})$, the weight of each particle is calculated by:

(41) $$w_{k}^{i}=w_{k-1}^{i}\frac{p(y_{k}|x_{k}^{i})p(x_{k}^{i}|x_{k-1}^{i})}{q(x_{k}^{i}|x_{k-1}^{i},y_{k})}$$

Then the maximum weight $w_{k-\max}^{i^{*}}$ and the minimum weight $w_{k-\min}^{i^{*}}$ is recorded, and the observation innovation $y_{k-\max}^{i}$, $y_{k-\min}^{i}$, and Euclidean distance $L_{\max}^{}$ and $L_{}^{i}$ can be calculated, which are expressed as:

(42) $$L_{\max}={\left(y_{k-\min}^{i}-y_{k-\max}^{i}\right)}^{T}\cdot (y_{k-\min}^{i}-y_{k-\max}^{i})$$

(43) $$L^{i}={\left(y_{k}^{i}-y_{k-\max}^{i}\right)}^{T}\cdot (y_{k}^{i}-y_{k-\max}^{i})$$

where $y_{k-\max}^{i}=y_{k}-h(x_{k-\max}^{i})$, $y_{k-\min}^{i}=y_{k}-h(x_{k-\min}^{i})$, $x_{k-\max}^{i}$ and $x_{k-\min}^{i}$ represent the particles that correspond to the maximum weight and the minimum weight.

The weight of particles can be modified as:

(44) wki*=wki*+(wk−maxi*N)⋅sin(LiLmax⋅π2)⋅β

where β is the adaptive coefficient determined by the statistical characteristics of observation noise, and:

(45) β={Kα′α′≤ε0α′>ε

where α′ is the dilution of precision that reflects statistical characteristics of observation noise. By changing the size of α′, we can adaptively adjust the distribution of weights and increase the weights of useful particles. When α′ is large, the observation accuracy is low, otherwise, the observation accuracy is relatively high. ε is the threshold that determined by experience, K is the proportional constant, and Kα′>0. When the observation noise is small, let β=0, that is, do not adjust the likelihood distribution. Otherwise, when the likelihood distribution is in the peak or at the tail of the transfer prior distribution, let β=Kα′, that is, artificially make the likelihood distribution wider.

## 5. Simulation Experiment and Result Analysis

In this section, we compare the navigation errors of the proposed SINS/SRS/GNS autonomous navigation system with the SINS, SINS/SRS subsystem and SINS/GNS subsystem, respectively, by using the proposed RACDPF algorithm, to verify the performance of our SINS/SRS/GNS autonomous navigation system. Furthermore, under the same conditions, the simulation results of UKF, PF, and the proposed RACDPF are also compared to verify the performance of the proposed filtering algorithm, including comparisons of the accuracy, real-time and robustness of the filtering algorithms.

**Figure 3:**
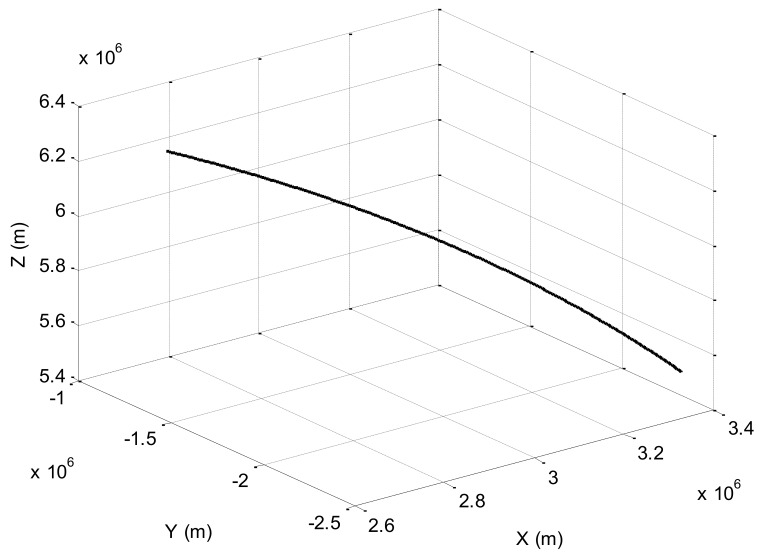
Flight trajectory.

In the experiment, the J2000 geocentric equatorial inertial coordinate system is selected for the coordinate system. Assume that the experimental data are from a flight of a spacecraft. The orbit parameters of the spacecraft are described as Semimajor axis 6947.035365 km, Eccentricity 0.001088, Orbit inclination 22.998°, Right ascension of ascending node 334.87°, Argument of perigee 341.452°, True anomaly 231.43°. We selected a part of the flight, which lasted for 1500 s, and the initial position is (3,330,812, −2,488,259, 5,565,647). The flight trajectory is shown in Figure 3.

In the simulation process, the initial alignment error of SINS is 0, the initial velocity error is 1 m/s, the initial position error is 10 m, and the initial attitude error is $10^{\prime \prime }$. The parameter for adaptive factor calculation is c=1.5 [25,27]. The number of particles is M=200, the simulation time is 1500 s, and the filtering period is 1 s. The parameters of the sensors used in the simulation are shown in Table 1.

(1) Simulation verification of the subsystems

The simulation of SINS, SINS/SRS subsystem and SINS/GNS subsystem are carried out respectively, and the results are compared to verify the performance of the subsystems.

**Figure 4:**
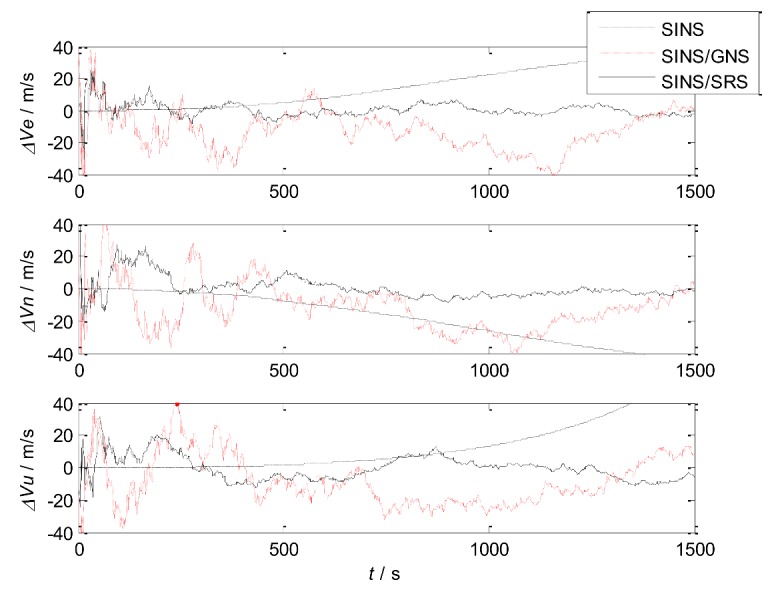
Velocity errors of the subsystems.

**Figure 5:**
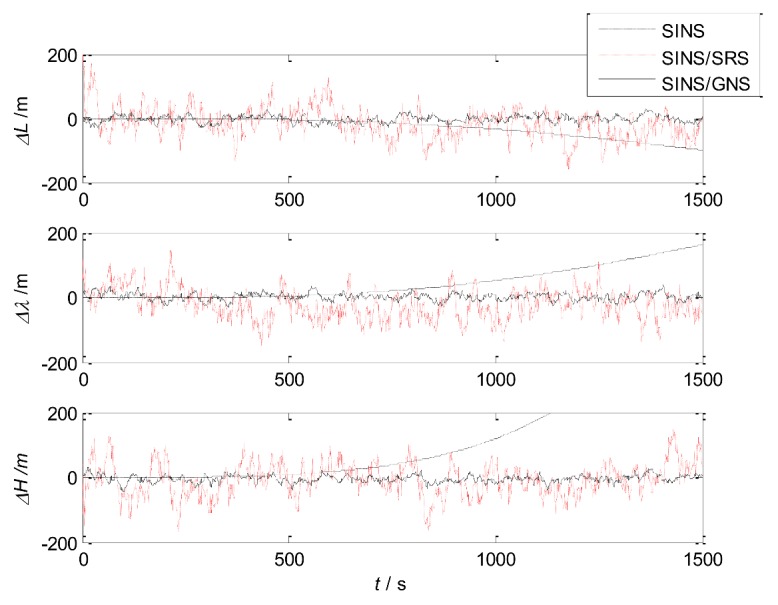
Position errors of the subsystems.

The simulation results are shown in Figure 4 and Figure 5. The error statistics of the subsystems are shown in Table 2.

It can be seen that the velocity error and position error of SINS accumulate with time and diverge, which cannot meet the high accuracy requirement of navigation system. Therefore, other navigation methods need to be used to correct SINS.

For the SINS/GNS subsystem, GNS can obtain high position accuracy, so it is mainly used to correct the position error of SINS. But the velocity accuracy is poor, and the correction effect is not obvious. For the SINS/SRS subsystem, SRS can obtain good velocity accuracy, so the velocity accuracy after correction is high, but the position accuracy is poor. Therefore, single SINS/GNS or SINS/SRS subsystem cannot meet the needs of autonomous navigation and positioning. We need to combine both of them to design the SINS/SRS/GNS autonomous integrated navigation system to improve the navigation accuracy.

(2) Simulation verification of the autonomous integrated navigation system

**Figure 6:**
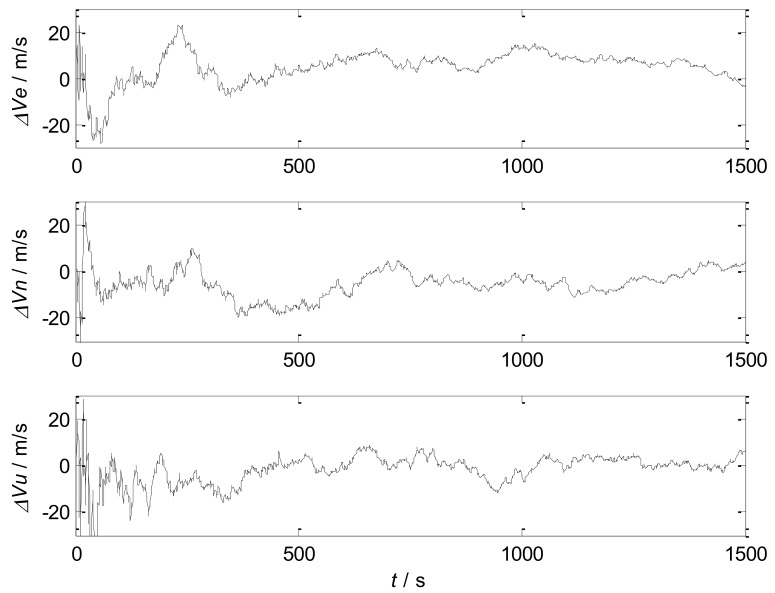
Velocity error of the autonomous integrated navigation system.

**Figure 7:**
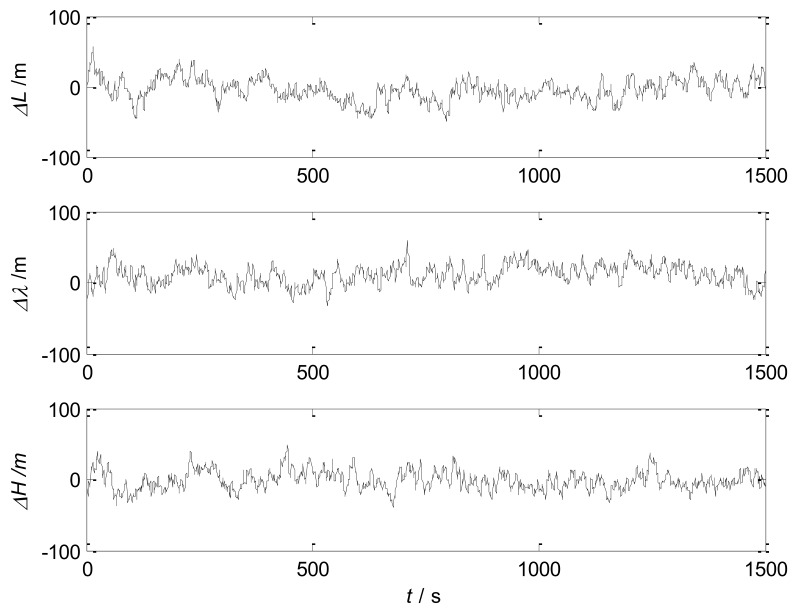
Position error of the autonomous integrated navigation system.

The simulation of SINS/SRS/GNS autonomous integrated navigation system is carried out, and the results are shown in Figure 6 and Figure 7. Table 3 shows the error statistics of the autonomous integrated navigation system.

The simulation results show that the designed SINS/SRS/GNS autonomous integrated navigation system adopts information fusion technology to combine SINS with SRS and GNS, and complements the three systems in performance. Therefore, the SINS/SRS/GNS system overcomes the defects of single navigation system, and exhibits a good performance. Therefore, the designed SINS/SRS/GNS system effectively improve the accuracy and reliability of autonomous navigation systems.

(3) Performance verification of filtering algorithm

Based on the above performance comparison and analysis, UKF and PF are also applied to the SINS/SRS/GNS autonomous integrated navigation system for filtering respectively, in order to verify the performance of the proposed RACDPF algorithm and autonomous integrated navigation system. We will compare and analyze the accuracy, real-time and robustness of the nonlinear filtering algorithms (UKF, PF and RACDPF) respectively, so as to evaluate the performance of the filtering algorithms from more aspects.

**A. Accuracy Comparison of Filtering Algorithms**

**Figure 8:**
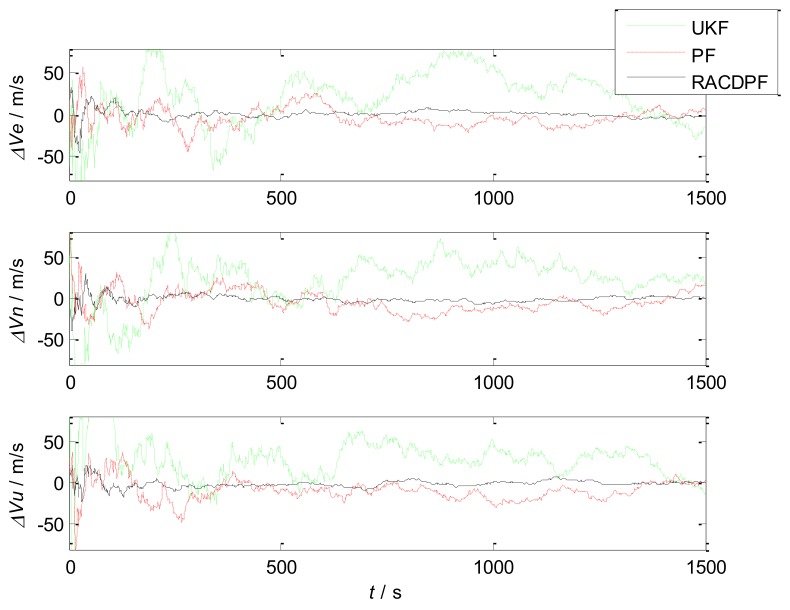
Velocity errors of the three filtering algorithms (M=200).

**Figure 9:**
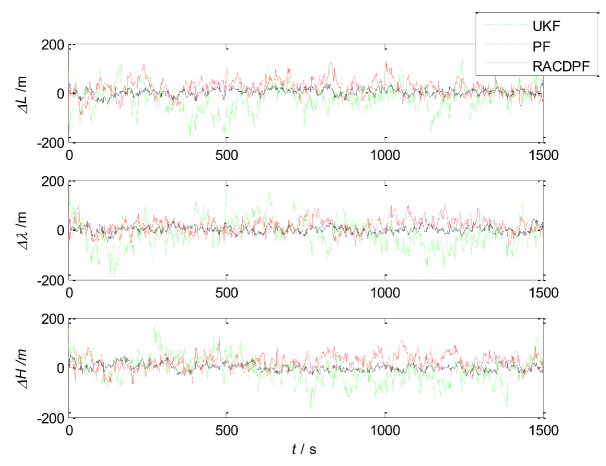
Position errors of the three filtering algorithms (M=200).

We define the filtering accuracy as the difference between the estimated state value and the reference state value. The accuracy of the nonlinear filtering algorithms is compared and analyzed by the following steps:

(1) Use UKF, PF and RACDPF for filtering and then calculate the velocity error and position error of each filtering algorithm. The results are shown in Figure 8 and Figure 9. In order to facilitate statistical results, the position error can be defined as:
  (46) $$\delta p=\sqrt{{\left(\delta L\right)}^{2}+{\left(\delta \lambda \right)}^{2}+{\left(\delta h\right)}^{2}}$$
  the velocity error can be defined as:
  (47) $$\delta V=\sqrt{{\left(\delta V_{E}\right)}^{2}+{\left(\delta V_{N}\right)}^{2}+{\left(\delta V_{U}\right)}^{2}}$$
  Then, the error statistics of the three filtering algorithms can be obtained, as shown in Table 4.
(2) Repeat Step (1) in the case of particle number M=50 and M=500 respectively, and the results are shown in Table 5 and Table 6.

It can be seen that the accuracy of UKF is the worst. PF inevitably displays a particle degradation phenomenon after several iterative computations, therefore the filtering accuracy is limited. RACDPF uses the robust adaptive factor to control the kinematic model information and observation model information, for suppressing the influence of abnormal interference, thus the filtering accuracy is better than that of UKF and PF. From Table 4, Table 5 and Table 6, it can be seen that the number of particles can obviously affect the accuracy of PF and RACDPF because PF uses samples to approximate the a priori information and a posteriori information, and the more the number of samples, the closer to the true distribution.

**B. Real-Time Comparison of Filtering Algorithms**

The factors that affect the real-time performance of the filtering algorithm include algorithm complexity, filtering condition and hardware processing ability. The algorithm complexity is the main parameter to describe the degree of difficulty in algorithm implementation. In this section, The real-time performance of UKF, PF and RACDPF is researched under the same hardware platform, software platform and initial conditions.

The equivalent computational complexity and running time of each filtering algorithm are are shown in Table 7, where M is the number of particles, n is the state dimension, O(·) represents the computational complexity of each recurrence calculation for the algorithms.

It can be seen that PF and its improved algorithms need to sample a large number of particles, allocate weights and resample, which is complex and computationally burdensome, therefore the algorithm running time is obviously higher than that of UKF. Therefore, the number of particles should be determined according to the specific application requirements.

**C. Robustness Comparison of Filtering Algorithms**

For a given filtering algorithm, its robustness is manifested in the performance that when the system parameters or the external environment change, the filtering algorithm can still maintain certain filtering accuracy.

In order to verify the robustness of the filtering algorithms (UKF, PF and RACDPF), the errors of variance 1 m^2^/s^2^ and 4 m^2^ are added to the velocity and position observations of the experimental data, respectively. Then, the position RMSEs of the two sets of experimental data are calculated by each filtering algorithm, respectively. The number of particles *M* = 200, and the other conditions are consistent with the previous. The results are shown in Table 8.

The effect of abnormal disturbances on the UKF and PF is more significant than RACDPF, this is because UKF and PF cannot deal with abnormal interferences. However, RACDPF can control the kinematic model information and the observation model information by selecting the appropriate robust adaptive factor to suppress the influence of abnormal interferences. Therefore, the RACDPF is the least affected.

The effect of abnormal disturbances on the UKF and PF is more significant than RACDPF, this is because UKF and PF cannot deal with abnormal interferences. However, RACDPF can control the kinematic model information and the observation model information by selecting the appropriate robust adaptive factor to suppress the influence of abnormal interferences. Therefore, the RACDPF is the least affected.

## 6. Conclusions

In order to satisfy the requirements of the high precision and reliability of the autonomous navigation system, a new SINS/SRS/GNS autonomous integrated navigation method is proposed in this paper. The scheme of the SINS/SRS/GNS autonomous integrated navigation system is designed, and the corresponding filtering algorithm, a robust adaptive central difference particle filtering (RACDPF) algorithm, is also proposed. This algorithm can control the state model information and observation model information, suppress the influence of abnormal interference, and adaptively adjust the weight of particles after importance sampling, thus improving filtering accuracy. The simulation results show that the proposed method possesses high accuracy, strong robustness and good reliability, and can satisfy the performance requirements of autonomous navigation in a certain degree. Because of the abundant natural celestial bodies and their spectral redshift information in space, the SINS/SRS/GNS autonomous integrated navigation method can be applied to spacecraft, space station, and satellite orbit determination, thus providing a new solution for the autonomous navigation technology.

## Figures and Tables

**Table 1:** Parameters of the sensors used in the simulation.

Parameter Type	Value
Gyro constant drift	0.02°/h
Gyro random drift	$0.005{}^{\circ}/\sqrt{h}$
Constant drift of accelerometer	0.05 mg
Random drift of accelerometer	$0.005\;\mathrm{mg}/\sqrt{h}$
Accuracy of geomagnetic matching	20 m
Accuracy of velocity measurement with SRS [31]	5.5 m/s

**Table 2:** The error statistics of the subsystems.

Errors		The Subsystems	
		SINS/GNS	SINS/SRS
Velocity RMSE (m/s)	East	9.4491	5.3219
	North	8.7579	5.0692
	Up	8.6688	5.2851
Position RMSE (m)	Longitude	16.8274	65.6695
	Latitude	17.3205	68.7935
	Height	16.7775	70.1250

**Table 3:** The error statistics of the autonomous integrated navigation system.

	Error		Value
SINS/SRS/GNS autonomous integrated navigation system	Position RMSE(m)	Longitude	17.0375
		Latitude	16.3565
		Height	16.7865
	Velocity RMSE(m/s)	East	5.3698
		North	5.1550
		Up	5.2546

**Table 4:** The error statistics of the three filtering algorithms (M=200).

Filtering Algorithm	Velocity Error/m/s	Position Error/m
UKF	23.8122	56.7445
PF	11.1434	33.4074
RACDPF	5.1529	16.1745

**Table 5:** The error statistics of the three filtering algorithms (M=50).

Filtering Algorithm	Velocity Error/m/s	Position Error /m
UKF	23.8122	56.7445
PF	15.2567	39.9238
RACDPF	6.1740	18.2510

**Table 6:** The error statistics of the three filtering algorithms (M=500).

Filtering Algorithm	Velocity Error/m/s	Position Error/m
UKF	23.8122	56.7445
PF	10.9682	28.4450
RACDPF	4.5424	13.5341

**Table 7:** Real-time performance of the nonlinear filtering algorithms.

Filtering Algorithm	Computational Complexity	Running Time/s		
		M=50	M=200	M=500
UKF	$O(n^{4})$	0.6728	0.6728	0.6728
PF	$O(Mn^{3})$	1.0795	2.4141	4.8626
RACDPF	$O(Mn^{3})$	1.1589	2.6979	5.0032

**Table 8:** Robustness performance of the nonlinear filtering algorithms.

Filtering Algorithm	Position RMSE/m		
	Errors Are Added	No Error Is Added	Difference
UKF	61.0342	56.7445	8.2897
PF	38.8110	33.4074	5.4036
RACDPF	17.3852	16.1745	1.2107
